# Calcium and cAMP directly modulate the speed of the *Drosophila* circadian clock

**DOI:** 10.1371/journal.pgen.1007433

**Published:** 2018-06-07

**Authors:** Angelina Palacios-Muñoz, John Ewer

**Affiliations:** Centro Interdisciplinario de Neurociencia de Valparaíso, Facultad de Ciencias, Universidad de Valparaíso, Gran Bretaña 1111, Valparaíso, Chile; Washington University in Saint Louis School of Medicine, UNITED STATES

## Abstract

Circadian clocks impose daily periodicities to animal behavior and physiology. At their core, circadian rhythms are produced by intracellular transcriptional/translational feedback loops (TTFL). TTFLs may be altered by extracellular signals whose actions are mediated intracellularly by calcium and cAMP. In mammals these messengers act directly on TTFLs via the calcium/cAMP-dependent transcription factor, CREB. In the fruit fly, *Drosophila melanogaster*, calcium and cAMP also regulate the periodicity of circadian locomotor activity rhythmicity, but whether this is due to direct actions on the TTFLs themselves or are a consequence of changes induced to the complex interrelationship between different classes of central pacemaker neurons is unclear. Here we investigated this question focusing on the peripheral clock housed in the non-neuronal prothoracic gland (PG), which, together with the central pacemaker in the brain, controls the timing of adult emergence. We show that genetic manipulations that increased and decreased the levels of calcium and cAMP in the PG caused, respectively, a shortening and a lengthening of the periodicity of emergence. Importantly, knockdown of CREB in the PG caused an arrhythmic pattern of eclosion. Interestingly, the same manipulations directed at central pacemaker neurons caused arrhythmicity of eclosion and of adult locomotor activity, suggesting a common mechanism. Our results reveal that the calcium and cAMP pathways can alter the functioning of the clock itself. In the PG, these messengers, acting as outputs of the clock or as second messengers for stimuli external to the PG, could also contribute to the circadian gating of adult emergence.

## Author summary

Circadian clocks impose daily periodicities to animal behavior and physiology. At their core, circadian rhythms are produced by intracellular transcriptional/translational feedback loops (TTFL). TTFLs may be altered by extracellular signals whose actions are mediated intracellularly by calcium and cAMP. In *Drosophila*, calcium and cAMP levels affect the periodicity of *Drosophila* circadian rhythms, but whether this is due to direct actions on the TTFLs themselves or is a consequence of changes induced to the complex interrelationship between different classes of central pacemaker neurons is unclear. Here we used the non-neuronal circadian clock located in the prothoracic gland (PG) to show that these messengers affect the speed of the circadian clock that controls the timing of adult emergence and suggest that these actions are mediated by CREB. Importantly, since calcium and cAMP are also output signals of the clock, they may contribute to the mechanism that imposes a circadian gating to the timing of adult emergence.

## Introduction

Circadian clocks impose a daily rhythmicity to the behavior and physiology of multicellular organisms. In animals, the circadian system consists of a principal circadian pacemaker located in the central nervous system (CNS) as well as of autonomous circadian pacemakers located in most peripheral tissues. At their core, circadian rhythms are produced by intracellular transcriptional/translational feedback loops (TTFL) [1,2], and coordination at different levels ensures that the organism express a unified circadian time. At the level of the central pacemaker, a number of transmitters [3–10] and neuropeptides [11–16] mediate the production of coherent network-wide circadian oscillations. In addition, central and peripheral clocks are coordinated, maintaining a stable phase relationship. In mammals this is accomplished through a variety of channels that are still poorly understood, and which include electrical, endocrine, metabolic, and even thermal signaling [17]. In insects, most peripheral clocks are synchronized with the central pacemaker by entrainment to a common signal (e.g., light, which can penetrate the translucent exoskeleton, or temperature; [18]), with the notable exception of the clock in the non-neuronal prothoracic gland (PG) clock, which is coupled to the brain clock by neuropeptide action [19]. In addition to intra- and inter- pacemaker coordination, clocks can also be entrained by external stimuli such as light, which is a powerful entraining signal, as well as by an organism’s physiological and behavioral state [1, 20]. For example, in mammals the liver clock can be entrained by daily feeding, which retains a phase difference with the central clock once restricted feeding ends [21].

Coordination of circadian activity within the mammalian central pacemaker and the phase-shifting effects of light are mediated intracellularly by calcium and cAMP, which ultimately funnel their actions through the calcium/cAMP-dependent transcription factor, CREB, which acts on CRE (calcium/cAMP-dependent response) elements to change clock gene expression [22–26]. In insects, a variety of circumstantial evidence suggests that calcium and cAMP can alter the functioning of the intracellular transcription/translation feedback loop (TTFL) of central pacemaker neurons [26]. For example, chronic buffering of calcium within pacemaker cells causes a progressive lengthening of the circadian periodicity of adult locomotor activity [27] and mutations that alter cAMP levels [28] or CREB function [29] alter the fly’s free-running period; likewise, acute changes in pacemaker excitability cause phase shifts in locomotor activity rhythms, which may be mediated by CREB [30]. Nevertheless, evidence for a direct role for calcium, cAMP, and CREB in pacemaker TTFL function is lacking. Indeed, calcium is involved in synaptic transmission and is the second messenger for some circadian neuropeptides (e.g., sNPF [19]) and cAMP is the second messenger for the principal circadian transmitter PDF (Pigment-Dispersing Factor) in *Drosophila* [31–33]. Thus, calcium and cAMP could alter TTFLs circadian clock output indirectly, by changing the complex actions and interactions between different classes of pacemaker neurons, which, for instance, differ in their phasing of calcium oscillations yet share the same timecourse of clock gene expression [34]. For this reason we investigated the function of calcium and cAMP on the functioning of the non-neuronal peripheral PG clock.

The PG is a peripheral endocrine gland that produces the molting hormone ecdysone. It contains a clock that is coupled to the central pacemaker and restricts the time of emergence (eclosion) of the adult fly to the early part of the day [19,35,36]. We investigated the role of calcium and cAMP on circadian clock function by determining the consequences on the rhythmicity of emergence of genetically manipulating the levels of these second messengers. We found that increasing or decreasing the levels of calcium and cAMP in the PG caused, respectively, a shortening and a lengthening of the periodicity of emergence. Importantly, knockdown of CREB in the PG caused an arrhythmic pattern of eclosion. Interestingly, the corresponding manipulations directed at central pacemaker neurons had similar effects on the rhythmicity of eclosion and of adult locomotor activity, suggesting a common mechanism. Direct measurements of calcium and cAMP levels in the PG revealed that these vary during the course of the day. Since calcium and cAMP are second messengers of many transduction pathways, our findings suggest that calcium and cAMP could provide a pathway through which different stimuli, both internal and external to the clock, could alter the period of the circadian clock. Furthermore, since calcium and cAMP play an important role in ecdysone synthesis and secretion, our findings raise the possibility that calcium and cAMP, produced as outputs of the clock or as second messengers for stimuli external to the PG, could contribute to the gating of adult emergence.

## Results

### Manipulating calcium levels in the PG cells affects the periodicity of adult emergence

**Fig 1:**
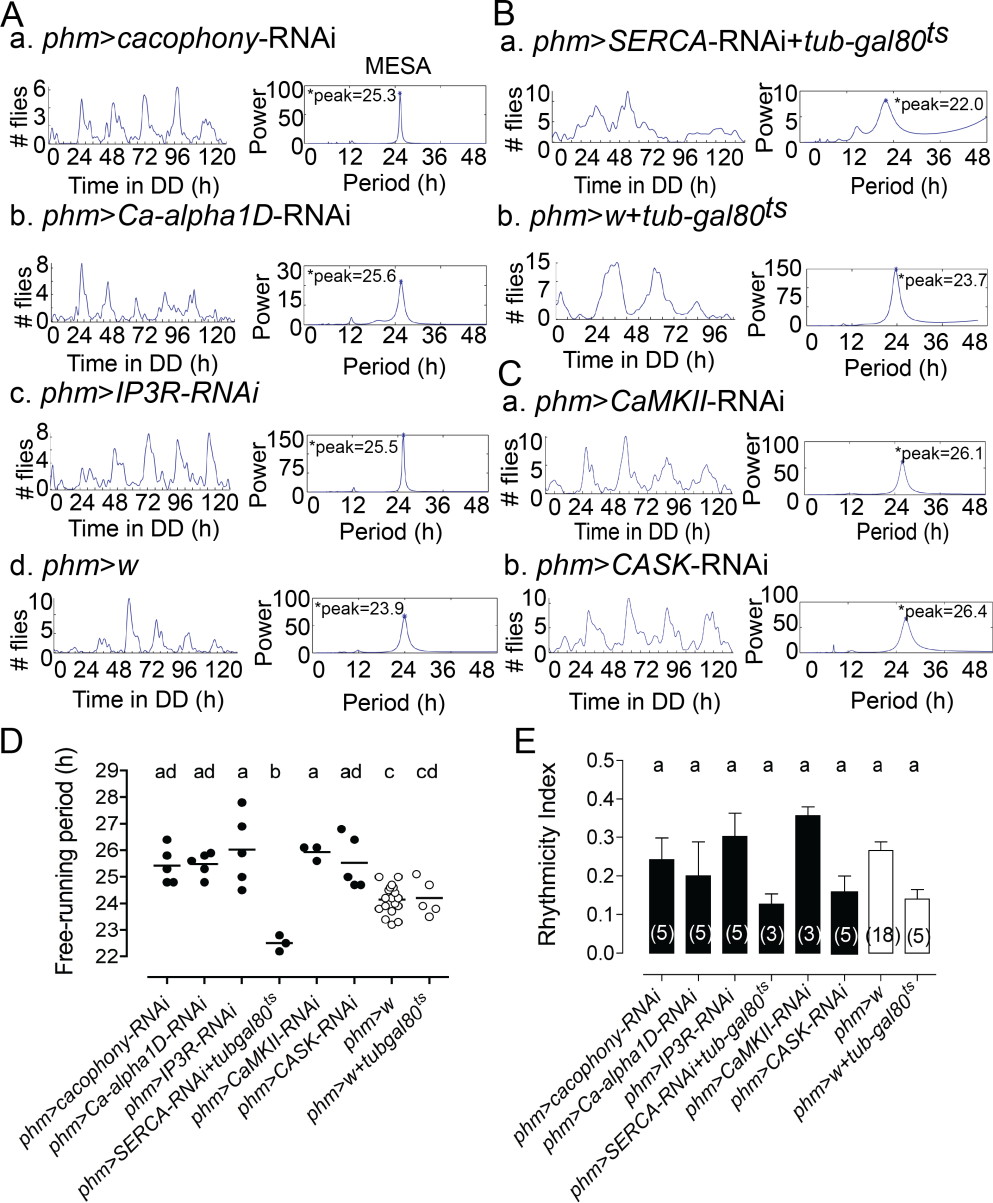
Knockdown of genes in calcium pathway in the PG affects the periodicity of eclosion. (A) Knockdown of calcium channels *cacophony* (a), *Ca-alpha1D* (b), and of IP3 receptor (*IP3R*) (c) in the PG lengthen the periodicity of eclosion with respect to controls (d). Records show time course of emergence of a single population in DD (left) and corresponding MESA analyses (right); principal periodicity is indicated. (B) Knockdown of *SERCA* in the PG shortens the periodicity of eclosion (a) compared to control (b). (C) Knockdown of *CaMKII* (a) and *CASK* (b) in the PG lengthens the periodicity of eclosion. (D) Free-running periodicity for results shown in A-C. Each point indicates results from separate experiments; average is indicated by horizontal line; different letters indicate statistically different groups (*p*<0.05; one-way ANOVA, Tukey’s *post hoc* multiple comparison analyses). (E) Average rhythmicity index (RI) values (± SEM) for results shown in A-C. Numbers in parenthesis indicate number of separate experiments; different letters indicate statistically different groups (*p*<0.05; one-way ANOVA, Tukey’s *post hoc* multiple comparison analyses). In A-C, GAL4 driver was used in combination with UAS-*dcr2* to potentiate effectiveness of RNAi knockdown. Flies bearing only UAS-RNAi transgenes express normal rhythmicity of emergence (S1 Fig).

**Fig 2:**
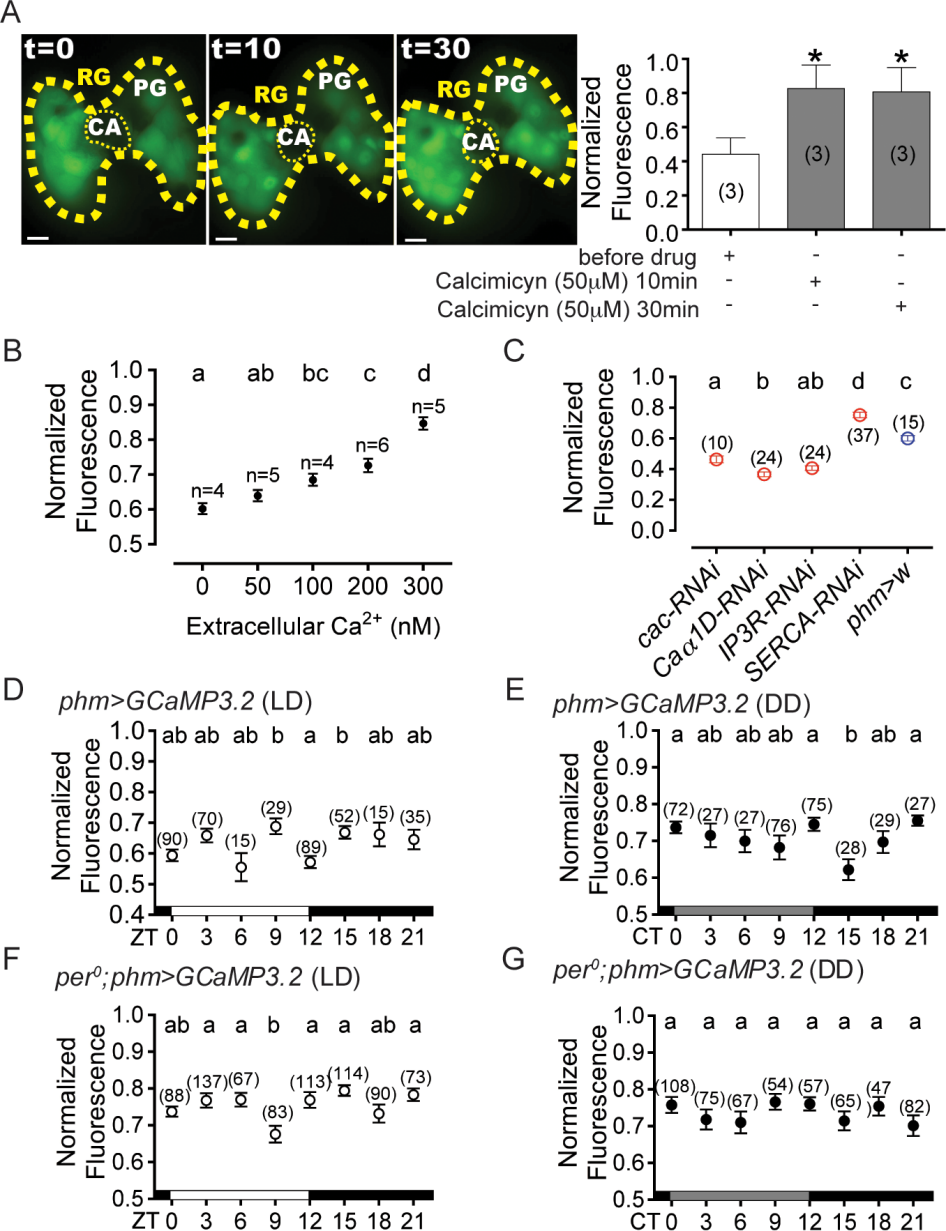
Calcium levels in the PG vary during the course of the day. (A) *Left*: Representative images of GCaMP fluorescence in the PG before (t = 0), and 10 min and 30 min after incubation with 50μM calcimycin (external [calcium]: 1mM) and *Right*: quantitation of the fluorescence in the PG; numbers in parenthesis indicate number of PG glands measured; *p<0.05 (paired *t*-test compared with signal measured before adding drug). (B) Dose response of GCaMP sensor measured using increasing extracellular calcium concentrations; n: number of PG cells (taken from at least 4 PGs per condition). (C) Levels of fluorescence in controls and in animals bearing knockdown of *calcium channels* (*cacophony* [*cac*] and *Ca-alpha1D*), *IP3* receptor (*IP3R*) and *SERCA* in the PG (using *phm*-GAL4 driver)(all measured at ZT12). The number of cells analyzed is indicated above colored circle. For each experiment 4–8 animals were examined. Error bars denote SEM. (D-G) Levels of fluorescence in the PG of wildtype animals at different times of day under LD (D) and DD (E) conditions, and in the PG of *per*^0^ mutants under LD (F) and DD (G) conditions. Different letters indicate statistically significant differences (ANOVA with Tukey’s *post hoc* test). The number of cells is indicated in brackets and above each error bars. In A: PG: prothoracic gland, CA: *corpus allatum*, RG: ring gland. Scale bar 10μm; in A-G: Normalized fluorescence is quantitated in arbitrary units.

In order to investigate the role of calcium in regulating circadian clock function, we determined the consequences on the circadian rhythm of emergence of knocking down, specifically in the PG, genes involved in calcium homeostasis. As shown in Fig 1A, reducing the expression of two different calcium channels (*cacophony* and *Ca-alpha1D*; Fig 1Aa and 1Ab) and of the *IP3* (*Inositol 1*,*4*,*5*, *-tris-phosphate*) receptor (*IP3R*; Fig 1Ac) significantly lengthened the periodicity of adult emergence (Fig 1D), without affecting the strength of the rhythmicity (Fig 1E). By contrast, knockdown of *SERCA* (sarco/endoplasmic reticulum calcium ATPase) in the PG resulted in a significant shortening of the periodicity of eclosion (Fig 1Ba and 1D), also without significantly altering its associated rhythmicity index (Fig 1E). (In this case, *SERCA* was knocked down acutely at the end of metamorphosis using the GAL80^ts^ temperature-sensitive genetic system [37] because SERCA RNAi expression in the PG throughout life was lethal.) Such manipulations are expected to cause decreases and increases in calcium levels for *cacophony*, *Ca-alpha1D*, and *IP3R*, and *SERCA*, respectively, and direct measurement of calcium levels in the PG showed that this was indeed the case (cf. Fig 2C, below). We also probed the possible role of *CaMKII* (*calcium/calmodulin-dependent protein kinase I*) and *CASK* (*calmodulin-dependent protein kinase activity*) in the PG to determine if target proteins of calcium signaling might have an effect on the eclosion rhythm. As shown Fig 1C and 1D knockdown of *CaMKII* and of *CASK* in the PG (Fig 1Ca and Fig 1Cb, respectively) caused a significant lengthening of the periodicity of eclosion compared to that of the control (Fig 1D), without affecting strength of rhythmicity (Fig 1E). These results show that changing the levels of intracellular calcium affects the periodicity of the circadian clock. These changes can be effected by reducing calcium entry (e.g., through CACOPHONY or CA-ALPHA1D) or release from intracellular stores (e.g., via IP3), and could be mediated by CaMKII or CASK. Flies bearing single UAS-RNAi insertions for genes in calcium homeostasis pathway expressed normal circadian rhythmicity of eclosion (S1 Fig).

### Direct measurements of calcium in the PG

We measured intracellular calcium levels in the PG *in vivo* using the genetically-encoded sensor, GCaMP 3.2 (GCaMP) [38]. This sensor has been extensively used and causes no apparent cytotoxicity or behavioral defects [39]. In particular, it caused no defects in the circadian rhythmicity of eclosion (S2A–S2C Fig) or locomotor activity (S2D–S2F Fig) when expressed in the PG using the *phm*-GAL4 driver [19,40]. We determined the dynamic range and sensitivity of this sensor by incubating PGs with different concentrations of calcium in the presence of 50 μM calcimycin, a calcium ionophore (Fig 2A). As shown in Fig 2B, we found that the readout from the GCaMP sensor increased monotonically between 0 and 300nM calcium.

We then measured changes in calcium levels in the PG during the course of the day (and subjective day) at the beginning of metamorphosis (white pre-pupal stage, WPP). We chose this stage because the PG is intact this time and the circadian clock is fully functional [41], unlike in animals prior to emergence, when PG cells are undergoing apoptosis [42], which, in our hands, rendered such measurements variable and inconsistent. Overall, GCaMP readings were between 0.6 and 0.8, which corresponds to 50-300nM (see Fig 2B), and is consistent with published values. For instance, work done using *Manduca sexta* PG using Fura-2-loaded PG cells, reported a basal calcium concentration in the range of 50-200nM and maximal levels of 300-500nM following stimulation with the PTTH neuropeptide [43]. Importantly, genetic manipulations expected to increase and decrease the levels of calcium (expression in the PG of RNAi to *cacophony*, *Ca-alpha1D*, and *IP3R*, and RNAi to *SERCA*, respectively) did indeed cause the corresponding changes in GCaMP fluorescence (Fig 2C). This indicates that the GCaMP sensor can detect physiologically-relevant changes in intracellular calcium levels in the PG. We then measured the GCaMP signal at different times of day in the PG of wildtype animals of the same developmental age (WPP stage) under a 12h light: 12h dark regime (LD) (Fig 2D) and under conditions of constant darkness and temperature (DD)(Fig 2E). Frequency analyses of these data using JTK_cycle [44] and Lomb-Scargle [45] failed to reveal any significant circadian rhythmicity. Nevertheless, a more restricted ANOVA analysis revealed that calcium levels reached a minimum at 12h after lights on (beginning of the night; typically referred to as Zeitgeber (ZT) time 12, where ZT0 is lights-on) under LD conditions (Fig 2D), which was delayed about 3h under DD conditions (circadian time (CT) 15, early subjective night; where CT0 is the start of the subjective day)(Fig 2E). The absence of circadian rhythmicity in these data is surprising. However, calcium oscillations in the PG are known to be influenced by the brain [41]. Thus, it is possible that the timecourse we recorded in the PG reflects the sum of two circadian inputs, one driven by the brain, and the other produced by the PG itself. Our results contrast with those obtained previously, by Morioka and colleagues [41], which showed that baseline intracellular calcium concentrations in the PG reached a single minimum at the beginning of the day under LD condition. However, their readings were done every 6 hours and skipped the timepoint at which we observed an even lower (and minimal) level. In arrhythmic *per*^0^ mutants, we observed a single minimum under LD conditions at ZT9 (Fig 2F); we observed no significant changes under DD conditions (Fig 2G), as would be expected for an arrhythmic genotype.

### cAMP levels in the PG cells affect the periodicity of adult emergence

**Fig 3:**
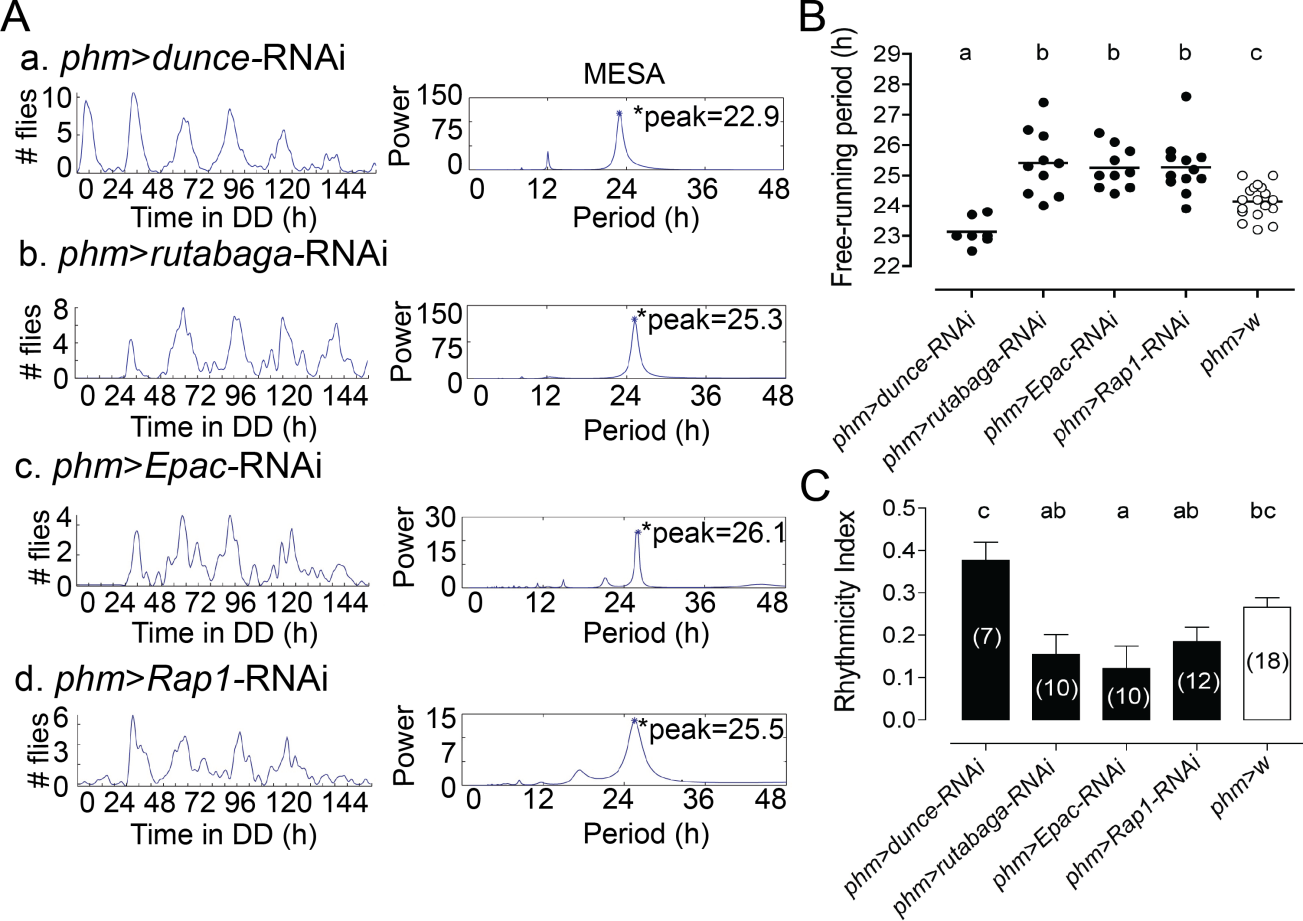
Knockdown in the PG of genes in cAMP pathway affects the periodicity of eclosion. (A) Knockdown of *dunce* in the PG shortens the periodicity of eclosion (a), whereas knockdown of *rutabaga* (b), *Epac* (c), and *Rap1* (d) in the PG lengthens the periodicity of eclosion, with respect to controls (B). (B) Free-running periodicity for results shown in (A). Each circle indicates results from separate experiments; average is indicated by horizontal line; different letters indicate statistically different groups (*p*<0.05; one-way ANOVA, Tukey’s *post hoc* multiple comparison analyses). (C) Average rhythmicity index (RI) values (± SEM) for results shown in (A). Numbers in parenthesis indicate number of separate experiments; different letters indicate statistically different groups (*p*<0.05; one-way ANOVA, Tukey’s *post hoc* multiple comparison analyses). Flies bearing only UAS-RNAi transgenes express normal rhythmicity of emergence (S3 Fig). In all experiments, GAL4 driver was used in combination with UAS-*dcr2* to potentiate effectiveness of RNAi knockdown.

**Fig 4:**
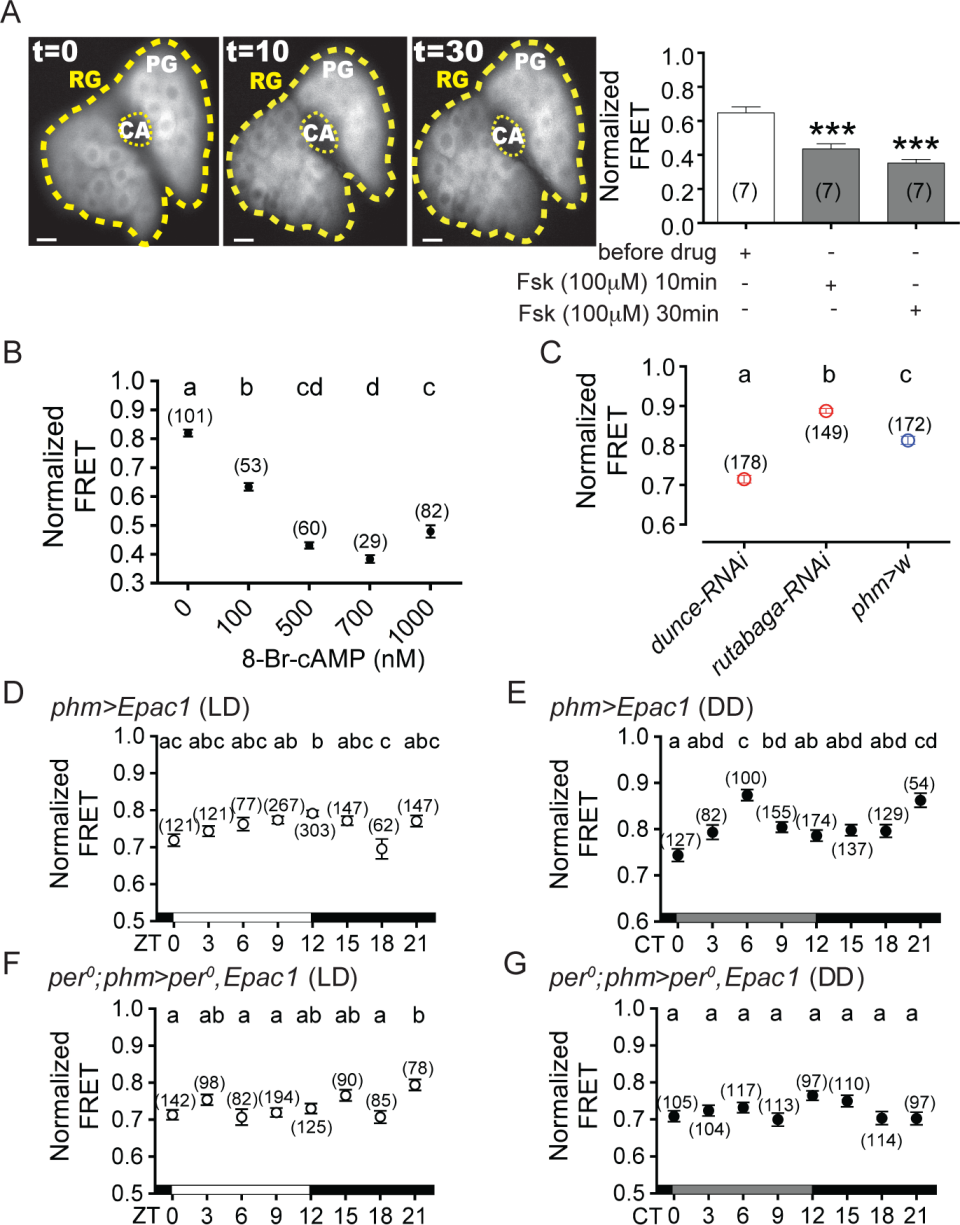
cAMP levels in the PG vary during the course of the day. (A) *Left*: Representative image of the signal from the cAMP sensor, *Epac1*, in the PG before (t = 0), and 10min and 30 min after incubation with 100μM forskolin (FSK); *right*: quantitation of the signal; numbers in parenthesis indicate number of glands measured. (B) Dynamic range of the *Epac1* sensor, determined using increasing concentrations of 8-Br-cAMP; n: number of prothoracic gland cells (taken from at least 4 PGs per condition). (C) cAMP levels in the PG in controls and in animals bearing knockdown of *dunce* and *rutabaga* in the PG (using *phm*-GAL4 driver)(all measured at ZT12). The number of cells analyzed is indicated above each circle. For each experiment 9–17 animals were examined. (D-G) cAMP levels in the PG of wildtype animals at different times of day under LD (D) and DD (E), and in *per*^0^ mutants under LD (F) and DD (G) conditions. Error bars denote SEM. In (A) ***p<0.0001 (paired *t*-test compared with signal measured prior to adding drug); scale bar, 10μm. In B-G, different letters indicate statistically significant values (ANOVA with Tukey’s *post hoc* test). The number of cells is indicated in brackets and above each error bars. In B-G: Normalized FRET signal is (YFP/CFP).

In parallel experiments we evaluated the role of cAMP in the control of the circadian clock that controls the timing of adult fly emergence. We found that knockdown of the cAMP phosphodiesterase (encoded by the *dunce* gene) in the PG shortened the periodicity of eclosion (Fig 3Aa and 3B), whereas knockdown in the PG of the calcium-dependent adenylate cyclase (encoded by the *rutabaga* gene), lengthened the periodicity of eclosion (Fig 3Ab and 3B), without affecting the strength of the rhythms (Fig 3C). Such manipulations are expected to cause increases and decreases in cAMP levels, respectively, and direct measurement of cAMP levels in the PG showed that this was indeed the case (cf. Fig 4C).

Furthermore, RNAi inhibition of others elements of the cAMP pathway in the PG also affected the periodicity of eclosion. Indeed, knockdown of *Epac* (*Exchange protein directly activated by cAMP*) and *Rap1* (*Ras-related protein 1* GTPase) in the PG caused a significant lengthening of the periodicity of eclosion (Fig 3Ac, 3Ad and 3B). Flies bearing single UAS-RNAi insertions for genes in cAMP pathway expressed normal circadian rhythmicity of eclosion (S3 Fig)

### cAMP levels vary in the PG cells during the course of the day

We next monitored cytoplasmic cAMP levels in the PG, using the genetically-encoded *Epac1 camps* cAMP sensor at the beginning of metamorphosis. This sensor has previously been used to measure cAMP levels in *Drosophila* neurons [31,33], and driving its expression in the PG did not affect the rhythmicity of eclosion (S4A–S4C Fig) or of locomotor activity (S4D–S4F Fig). Stimulating the PG with 100 μM forskolin (FSK), an activator of most forms of adenylyl cyclase [46], caused the *Epac1* signal to fall (as expected for a FRET-based sensor, for which an increase in cAMP would cause an decrease in signal) to a steady state level within 10 min (Fig 4A). In order to determine the sensitivity of this sensor, we measured the signal produced by increasing concentrations of 8-Br-cAMP, a membrane permeable analog of cAMP (Fig 4B). The FRET signal in the PG was highest for basal conditions, and decreased monotonically until 8-Br-cAMP concentrations of around 700nM. Importantly, genetic manipulations expected to increase and decrease the levels of cAMP (expression in the PG of RNAi of *dunce* and *rutabaga*, respectively) did indeed cause the corresponding changes in *Epac1* FRET signal, indicating that the *Epac1* sensor can detect physiologically-relevant changes in intracellular cAMP levels in the PG (Fig 4C).

We then measured the FRET signal in the PG at different times of day in wildtype animals of the same developmental age (WPP stage). As was the case for the calcium measurements, we did not detect any significant rhythmicity in the circadian range using the JTK_cycle [44] and Lomb-Scargle [45] spectral analyses. However, ANOVA analysis did reveal that under LD conditions cAMP levels were highest (lowest FRET signal) at ZT18, and lowest (highest FRET signal) around lights-off (ZT12)(Fig 4D). Under DD conditions highest cAMP levels were delayed by 3h (to CT0), whereas the lowest cAMP levels occurred at the middle of the subjective day (CT6)(Fig 4E). Finally, in arrhythmic *per*^0^ mutants, under LD conditions cAMP levels were highest at ZT18 and lowest around ZT21 (Fig 4F; similarly to rhythmic controls under LD conditions; Fig 4D); we observed no significant changes under DD conditions (Fig 4G), as would be expected for an arrhythmic genotype.

### Knockdown of CREB in the PG stops the PG clock

**Fig 5:**
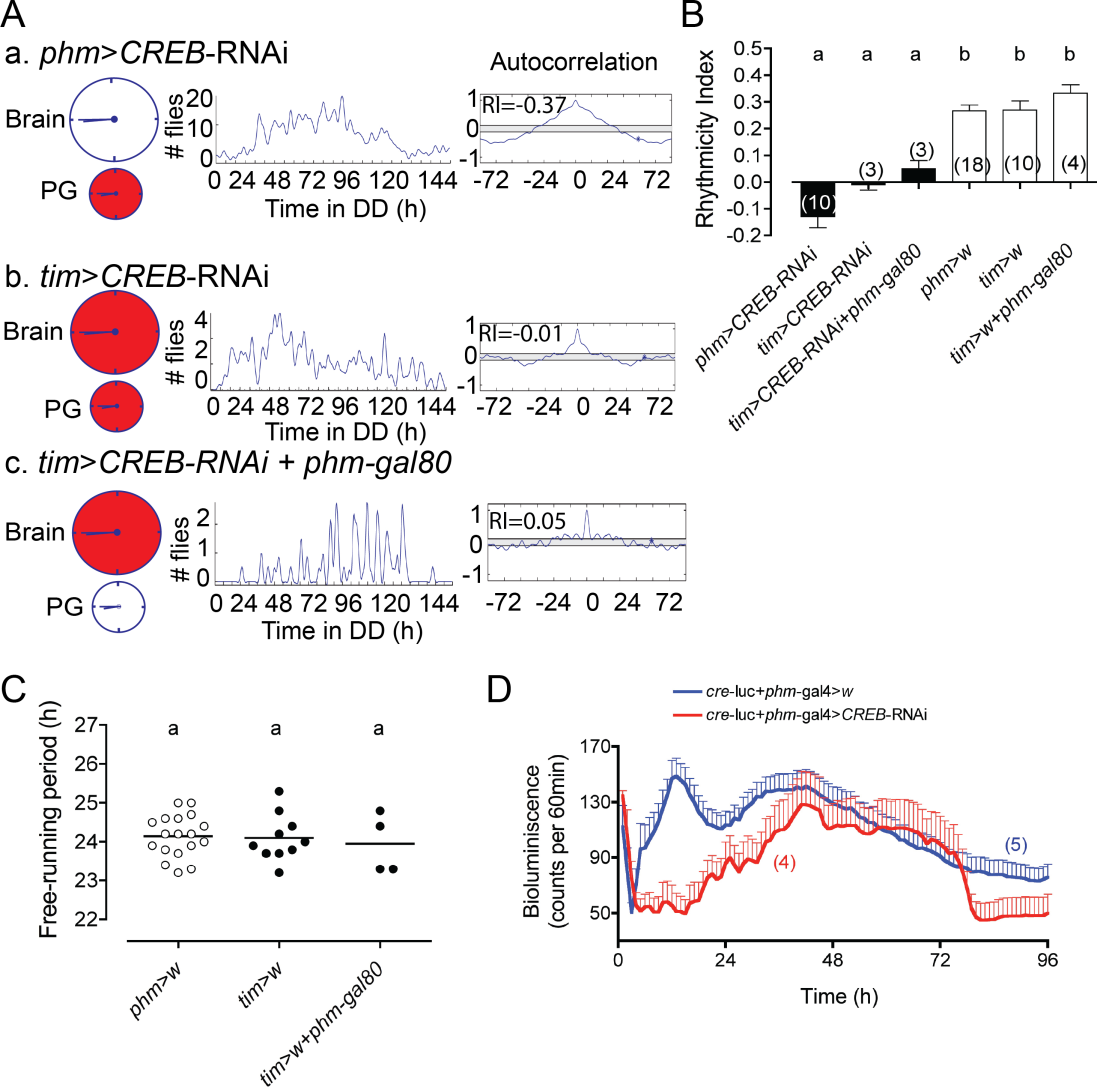
Knockdown of *CREB* in PG and/or central clocks causes arrhythmicity of eclosion. (A) Pattern of eclosion under DD following knockdown of *CREB* in the PG (a), in all clock cells (b), and in all clock cells except for the PG (c). Schematic shown on the left represents the brain and the PG clocks (large and small circle, respectively); red indicates where *CREB* was knocked down; middle: pattern of eclosion in DD; right: autocorrelation analysis of record, with dominant value of RI indicated. (B) Average rhythmicity index (RI) values (± SEM) for results shown in A and for controls; different letters indicate statistically different groups (*p*<0.05; one-way ANOVA, Tukey’s *post hoc* multiple comparison analyses). Numbers in parenthesis indicate number of separate experiments. (C) Free-running periodicity for controls (experimental groups, shown in A, were arrhythmic; cf., B). Each circle indicates results from a separate experiment; average is indicated by horizontal line; different letters indicate statistically different groups (*p*<0.05; one-way ANOVA, Tukey’s *post hoc* multiple comparison analyses). (D) Knockdown of *CREB* in the PG renders arrhythmic the pattern of *cre*-driven bioluminescence. Values plotted correspond to average ± SEM; numbers in parenthesis indicate number of records averaged.

The effects of manipulating calcium and cAMP levels in the PG on the periodicity of emergence suggest that these messengers could directly alter the clock TTFLs, and a variety of indirect evidence [30,47] suggests that they may act on the TTFL via CREB. As shown in Fig 5Aa and 5B, knockdown of CREB in the PG caused the expression of an arrhythmic pattern of emergence. Furthermore, using *cre*-luciferase as a reporter for clock output [29] showed that reducing CREB expression in the PG rendered this clock arrhythmic (Fig 5D). An arrhythmic pattern of emergence was also obtained when CREB was knocked down in all clock cells (Fig 5Ab and 5B), and when it was knocked down in all clock cells except for the PG clock (Fig 5Ac and 5B), compared to controls (Fig 5B and 5C) indicating that CREB plays a key role in all clock cells. Flies bearing transgenes for CREB RNAi for alone, and for the *phm*-GAL4 and *tim*-GAL4 drivers alone expressed normal circadian rhythmicity of eclosion (S3 Fig and S1 Fig, respectively).

### Knockdown of calcium and cAMP pathway in pacemaker cells of the brain affects the periodicity of adult emergence

**Fig 6:**
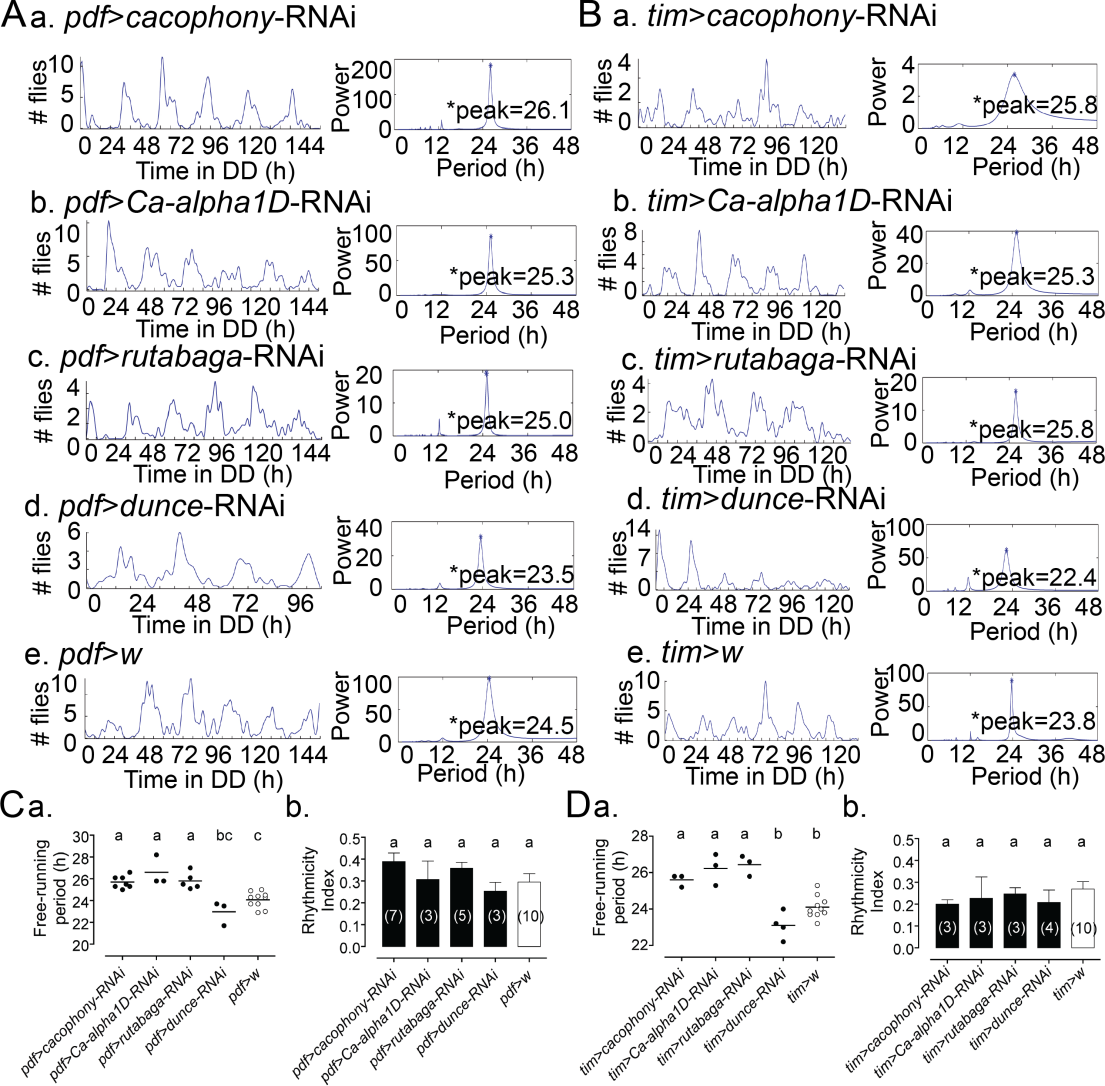
Knockdown of calcium and cAMP pathway in central clock affects the periodicity of adult emergence. (A)(a-e) *Left*: Pattern of emergence in DD following knockdown of *cacophony* (a), *Ca-alpha1D* (b), *rutabaga* (c), and *dunce* (d) in PDF neurons *versus* controls (e); *Right*: corresponding MESA analysis with value of free-running period (h) indicated. (B)(a-e) Left: pattern of emergence under DD of corresponding knockdown in all clock cells. (C) Free-running periodicity (a) and rhythmicity index (RI) values (b) for genotypes shown in (A) and for controls. In (a) each circle indicates results obtained in separate experiments; average is indicated by horizontal line; (b) shows the average (± SEM) RI, with different letters indicating statistically different groups (*p*<0.05; one-way ANOVA, Tukey’s *post hoc* multiple comparison analyses). (D) Free-running periodicity (a) and rhythmicity index (RI) values (b) for genotypes shown in (B) and for controls, represented as in (C). In all experiments, GAL4 driver was used in combination with UAS-*dcr2* to potentiate effectiveness of RNAi knockdown.

Our results show that manipulations of calcium and cAMP levels in the PG affect the rhythmicity of eclosion by directly affecting PG clock function, and that these actions may be mediated by CREB. In order to determine if a similar situation obtains for the central clock, we investigated the consequences on the circadian rhythmicity of eclosion of manipulating calcium and cAMP levels in central brain pacemaker neurons. Consistent with the results obtained for the PG, manipulations expected to decrease calcium (knockdown of calcium channels, *cacophony* and *Ca-alpha1D*) and cAMP levels (knockdown of *rutabaga*) in critical Small Ventral Lateral pacemaker neurons (sLNv) using the *pdf*-GAL4 driver, lengthened of the periodicity of eclosion (Fig 6Aa–6Ac and 6C). By contrast, knockdown of *dunce*, which is expected to increase the levels of cAMP in these pacemaker neurons, caused a shortening of the periodicity of eclosion (Fig 6Ad and 6C). Finally, similar manipulations using the *timeless*-GAL4 (*tim-GAL4*) driver, which is expressed in both central and peripheral pacemaker cells, produced similar results (Fig 6B and 6D). Flies bearing the *pdf*-gGAL4 driver alone expressed normal circadian rhythmicity of eclosion (S1 Fig). These results are comparable to the ones obtained following similar manipulations in the PG, and suggests that calcium and cAMP signaling plays a similar role in the central and the peripheral clock with respect to the circadian control of adult emergence.

### Knockdown of calcium and cAMP pathway in pacemaker cells of the brain affects the periodicity of locomotor activity of adult flies

**Fig 7:**
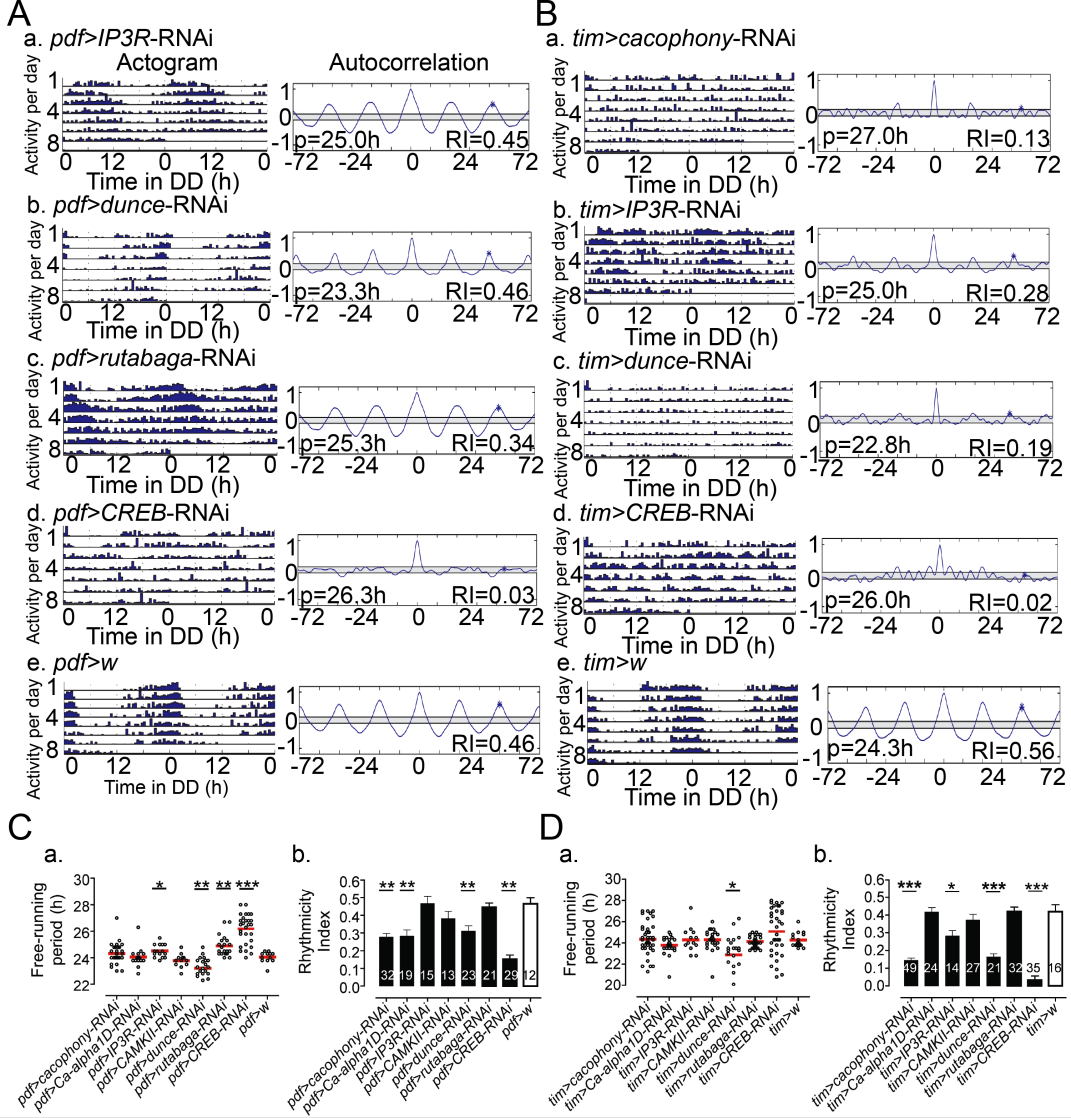
Knockdown of elements in calcium and cAMP pathway in clock cells affects the periodicity of adult locomotor activity rhythm. (A) *Left*: Representative record of adult locomotor activity under DD: following knockdown in PDF neurons of *IP3R* (a), *dunce* (b), *rutabaga* (c), *CREB* (d), and for *pdf*-GAL4 driver alone control (e); *Right*: corresponding Autocorrelation analyses with value of free-running period (h) and rhythmicity index (RI) indicated. (B) *Left*: Representative record of adult locomotor activity in DD: following knockdown in all clock cells of *cacophony* (a), *IP3R* (b), *dunce* (c), *CREB* (d), and for *tim*-GAL4 driver alone control (e). (C) Free-running periodicity (a) and rhythmicity index (RI) values (b) for genotypes shown in (A), plus those obtained for knockdown in PDF neurons of *cacophony*, *Ca-alpha1D* and *CAMKII*. In (a) each circle indicates individual flies tested; average is indicated by horizontal line; ***p<0.001; **p<0.01; *p<0.05 (two-tailed Student *t*-test *versus* control, with confidence interval of 95%). In (b) Average (± SEM) RI for genotypes shown in A-C(a), **p<0.01 (two-tailed Student *t*-test *versus* control, with confidence interval of 95%). Numbers indicate total number of flies tested. (D) Free-running periodicity (a) and rhythmicity index (RI) values (b) for genotypes shown in (B), plus those obtained for knockdown of *Ca-alpha-1D*, *CAMKII*, *rutabaga*, in all clock cells, represented as in (C). ***p<0.001; *p<0.05 (two-tailed Student *t*-test *versus* control, with confidence interval of 95%). In all experiments the GAL4 driver was used in combination with UAS-*dcr2* to potentiate effectiveness of RNAi knockdown.

Finally, we investigated the effects of manipulating calcium and cAMP levels in clock neurons on the circadian rhythm of locomotor activity of adult flies. As shown in Fig 7A and 7C and also summarized in Table 1, knockdown in sLNv neurons (using the *pdf*-GAL4 driver) of *dunce* shortened (Fig 7Ab, Fig 7C), whereas knockdown of *rutabaga* and *IP3R* lengthened (Fig 7Aa and 7Ac, Fig 7C), the periodicity of the locomotor activity rhythm. Previous work that manipulated calcium levels in sLNv by expressing buffer protein parvalbumin (PV) showed that PV overexpression eventually caused a lengthening of the free-running period [27]. Yet, this result was only visible in flies bearing several (>2) copies of the UAS-PV transgene and also, somewhat paradoxically, only after several weeks in DD. The relatively modest lengthening obtained when expressing *IP3R* RNAi in sLNv neurons, and the lack of detectable change following knockdown of *cacophony* and *Ca-alpha1D* (Fig 7Ca) may be due to low effectiveness of these RNAi lines in causing significant changes in calcium levels, and/or may require a longer monitoring period under DD conditions. Finally, and in line with previous work [27] knockdown of these genes in all clock cells (Fig 7B and 7D; Table 1) severely disrupted the free-running rhythms of locomotor activity. Similarly, knockdown of *CREB* in sLNv neurons (Fig 7Ad, Fig 7C) or in all clock neurons (Fig 7Bd, Fig 7D) caused the expression of an arrhythmic pattern of locomotor activity. Overall, these results suggest that calcium and cAMP levels affect clock function itself.

**Table 1:** Locomotor activity rhythmicity phenotypes following genetic manipulations of calcium and cAMP pathways in clock cells.

Genotype	N	% Rhythmicity	Period (h) ± SEM	RI ± SEM
*pdf*>*cacophony*-RNAi	32	100	24.3 ± 0.13	0.28 ± 0.02***
*pdf*>*Ca-alpha1D*-RNAi	19	95	24.1 ± 0.15	0.28 ± 0.04**
*pdf*>*IP3R*-RNAi	15	93	24.5 ± 0.13*	0.47 ± 0.04
*pdf*>*CaMKII*-RNAi	13	92	23.8 ± 0.14	0.38 ± 0.04
*pdf*>*dunce*-RNAi	23	100	23.2 ± 0.20**	0.31 ± 0.03**
*pdf*>*rutabaga*-RNAi	21	100	24.9 ± 0.17**	0.45 ± 0.02
*pdf*>*CREB*-RNAi	29	89	26.2 ± 0.22***	0.15 ± 0.02***
*pdf*>*w*	12	100	24.1 ± 0.13	0.47 ± 0.04
*tim*>*cacophony*-RNAi	49	71	24.4 ± 0.19	0.14 ± 0.01***
*tim*>*Ca-alpha1D*-RNAi	24	100	23.8 ± 0.16	0.41 ± 0.02
*tim*>*IP3R*-RNAi	14	93	24.3 ± 0.31	0.28 ± 0.03*
*tim*>*CaMKII*-RNA	27	96	24.3 ± 0.18	0.37 ± 0.03
*tim*>*dunce*-RNAi	21	100	22.9 ± 0.42*	0.16 ± 0.02***
*tim*>*rutabaga*-RNAi	32	100	24.1 ± 0.09	0.42 ± 0.03
*tim*>*CREB*-RNAi	35	49	25.1 ± 0.37	0.03 ± 0.02***
*tim*>*w*	16	100	24.3 ± 0.20	0.42 ± 0.04
*cacophony*-RNAi>*w*	17	100	23.9 ± 0.21	0.30 ± 0.04
*Ca-alpha1D*-RNAi>*w*	24	100	23.8 ± 0.06	0.50 ± 0.06
*IP3R*-RNAi>*w*	6	100	23.8 ± 0.16	0.33 ± 0.05
*CaMKII*-RNAi>*w*	19	100	23.8 ± 0.05	0.48 ± 0.02
*dunce*-RNAi>*w*	18	100	24.2 ± 0.30	0.41 ± 0.04
*rutabaga*-RNAi>*w*	16	100	24.0 ± 0.14	0.37 ± 0.04
*CREB*-RNAi>*w*	18	100	23.8 ± 0.12	0.44 ± 0.04

Periods were calculated using Autocorrelation analysis. (N) Total number of flies examined; (SEM) standard error; (RI) rhythmicity index. Flies with RI<0.1 were scored as arrhythmic. Bold denotes genotypes that are significantly different from their corresponding controls.

***p<0.001

**p<0.01

*p<0.05 (two-tailed Student *t*-test *versus* relevant control, with confidence interval of 95%).

## Discussion

### Role of calcium and cAMP in circadian TTFL

Mammalian and insect circadian clocks are produced by intracellular transcriptional/translational feedback loops (TTFL). In the case of neuronal circadian pacemakers, the activity of the cells that make up the pacemaker is then coordinated through transmitter and neuropeptide action. In the mammalian SCN, these messengers cause changes in the intracellular levels of calcium or cAMP and are known to directly affect the TTFL via CREB-mediated changes of transcription [24,48]. In *Drosophila*, by contrast, the relationship between changes in calcium and cAMP and the functioning of TTFLs is less clear. For example, different neurons within the central brain pacemaker exhibit distinctly different phases of calcium oscillation while expressing essentially identically-timed rhythms of clock gene expression [49]. This suggests that the pattern of neuronal activity of different fly pacemaker neurons may be an emergent property that depends on their interconnection. Likewise, use of a CRE-luc transgene to probe CREB expression in the *Drosophila* brain [50] showed that most neuronal clusters, including non-clock neurons, expressed a circadian rhythmicity of bioluminescence, suggesting that CREB could be part of the clock’s output mechanism.

Here we explored directly the relationship between calcium and cAMP levels and clock function using the simpler, non-neuronal, peripheral clock housed within the PG endocrine gland. We found small but significant changes in calcium and cAMP levels during the course of the day, which persisted under constant conditions, suggesting that these second messengers could be part of the output of the clock. These changes did not show a circadian rhythmicity. This contrasts with previously reported measurements of cAMP levels in the adult fly brain, which showed a single maximum [28] and may reflect the fact that the PG clock is a slave of the brain clock [19,35]. Importantly, however, we found that manipulations that increased or decreased the levels of calcium and cAMP in the PG caused, respectively, a shortening and lengthening of the periodicity of eclosion, suggesting that they may also affect clock TTFL function. In the case of calcium, these manipulations were effective when they targeted calcium channels as well as elements involved in calcium storage, revealing that both mechanisms could mediate changes in clock periodicity. Furthermore, PG-specific knockdown of CaMKII and CASK also affected clock period, suggesting that the effects of changes in calcium levels on the clock could be effected by phosphorylation. These results suggest that the effects on locomotor activity rhythmicity caused by reducing calcium levels in central neurons [27] could, at least in part, also be due to actions on the intracellular TTFLs. For the case of cAMP, our results similarly suggest that the shortened circadian periodicity of adult locomotor activity rhythmicity reported for *dunce* mutants [28], may be due to direct actions on the brain clock TTFL.

How might calcium and cAMP ultimately affect the *Drosophila* clock TTFL? Circumstantial evidence suggests that these second messengers may funnel their actions through the calcium/cAMP-dependent transcription factor, CREB, as occurs in the mammalian SCN [22–24, 26]. Indeed, the consequences of acute [30] and sustained [47] changes in pacemaker excitability affect TTFLs and are associated with changes in CREB expression. Here, we found that targeted knockdown of CREB in the PG caused a loss of circadian rhythmicity of eclosion, revealing that CREB plays a key role in the PG clock TTFL function; a similar result was obtained following CREB knockdown in the central clock, indicating that CREB may also be critical for neuronal clock TTFLs. Interestingly, overexpression of CREB-binding protein (CBP) in *timeless*-expressing cells causes an arrhythmic pattern of eclosion and locomotor activity as well as abnormal expression of CLOCK/CYCLE-induced clock genes [51], which suggest that CREB and CBP proteins may play a critical role in the control of the *period* transcriptional activity, consistent with the presence of CREB binding sites [29] in this gene. Overall, the picture that is emerging from a variety of studies is that calcium and cAMP are actors in the output of the clock, and also play an important role in translating, via CREB, the actions of stimuli external to the clock into changes in TTFL speed and/or phase. Nevertheless, we also found that knockdown of *Epac* in the PG also caused a significant lengthening of periodicity, which suggests a role for the Epac pathway in regulating circadian clock periodicity, as occurs in the SCN [23]. Although Epac can lead to the activation of CREB, it can also regulate MAP kinase signaling [52], and MAP kinase p38 knockdown in *Drosophila* clock neurons induces a lengthening of the periodicity of locomotor activity by altering the degree of phosphorylation of PER protein, which delays its translocation to the nucleus [53]. This suggests an additional mechanism through which cAMP could alter TTFL cycling.

### Other roles for calcium and cAMP in the PG: Possible crosstalk with molting hormone pathway

The circadian rhythm of *Drosophila* adult emergence depends on functional clocks in both the brain and the PG [19,35] and recent evidence shows that the brain transmits time information to the PG via the PTTH neuropeptide acting on its receptor, TORSO [19]. Yet, it is unclear how circadian rhythmicity is produced by this system, since PTTH expression does not show circadian rhythmicity in *Drosophila* [19,54,55] nor is there any evidence for daily oscillations in molting hormone titers during fly metamorphosis [56]. One possibility is that circadian rhythmicity of emergence is accomplished by fine-tuning the levels of ecdysone (the precursor of the active steroid, 20-hydroxyecdysone) production and/or secretion via calcium and cAMP. Although PTTH acts via the tyrosine kinase coupled receptor TORSO, calcium and cAMP have long been associated with PTTH transduction [57], and more recently, calcium has also been shown to play a critical role in ecdysone secretion from the PG [58]. Thus, changes in calcium and cAMP levels due to clock activity could impinge upon the PTTH transduction pathway, ultimately causing changes in the rate of fall of ecdysone titers, thereby delaying or accelerating the rate of metamorphosis and causing emergence to be confined to a specific window of time. Such a mechanism of gating would not require circadian oscillations in 20-hydroxyecdysone titers, but only subtle changes to the rate of fall of 20-hydroxyecdysone titers, and result in the relatively wide eclosion gate observed in this insect species. This mechanism implies that pathways external to the clock and PTTH transduction could also affect the pace of metamorphosis through cross-talk with the PTTH transduction pathway. The detection of complex patterns of calcium oscillations in the PG [58] suggests that much remains to be understood about the functioning of this critical endocrine organ.

## Materials and methods

### Ethics statement

This research was approved by the Bioethics and Biosecurity committees of the Universidad de Valparaíso, Chile.

### Fly rearing and stocks

*Drosophila* strains were raised on standard cornmeal media and, unless noted, were maintained at room temperature (20–22°C) on a 12/12 hours light/dark cycle. The following GAL4 drivers were used: *phm-*GAL4 [40], *tim*-GAL4 and *pdf*-GAL4 (generously provided by Paul Taghert, Washington University, USA). The *phm*-GAL80 strain has previously been described [19]. The UAS-GCaMP 3.2 calcium sensor was kindly provided by Julie Simpson (HHMI, Janelia Park, USA) and the UAS-*Epac1* sensor was obtained from Paul Taghert [33]. UAS-RNAi lines were obtained from the National Institute of Genetics, Japan (NIG), the Vienna *Drosophila* RNAi Center, Vienna, Austria (VDRC) and the Bloomington *Drosophila* Stock Center at Indiana University, Bloomington, USA (BL). Typically, a number of RNAi lines were tested and the most effective one was then used for the majority of the experiments. The lines used and their source is indicated below; the line chosen for the results reported here are indicated in bold: for *IP*_*3*_*R (Inositol 1*,*4*,*5*, *-tris-phosphate receptor*; CG1063): **BL#25937,** VDRC#6486; *Dmca1A (cacophony or Calcium-channel protein α*_*1*_ *subunit A*; CG1522): **BL#27244,** VDRC#104168; *Dmca1D (Calcium-channel protein α*_*1*_ *subunit D*; CG4894): **BL#25830**; *SERCA* (*sarco-endoplasmic reticulum calcium ATPase or Calcium ATPase 60A*; CG3725): **VDRC#4474,** VDRC#107446, BL#25928; *dCREB-A (cyclic-AMP response element binding protein A*; CG7450): **BL#31900,** VDRC#110650, BL#27648; *CASK (calmodulin-dependent protein kinase activity*; CG6703): **BL#27556,** BL#35309, BL#32857; *dCaMKII* (*Calcium/calmodulin-dependent protein kinase II*; CG18069): **BL#29401**; *dunce* (*cAMP phosphodiesterase* or *PDE4*; CG32498): **BL#27250**, *rutabaga* (*adenylyl cyclase* or *AC*: CG9533): **BL#27035,** NIG#9533R-1, VDRC#5569, VDRC#101759; *Rap1* (*Ras related protein1* GTPase; CG1956): **BL#29434**, VDRC#110757; *D-EPAC* (*Exchange protein directly activated by cAMP*; CG34392): **BL#29317**, VDRC#50372, VDRC#50373.

### Eclosion assays

Crosses (30–60 females + 15 males) were reared at 20°C under LD 12:12 (lights-on at noon). Resulting pupae were placed in Trikinetics eclosion monitors (Trikinetics, Inc., Waltham, MA, USA). The following day the lights were permanently turned off before lights-on and eclosion monitored for 7–8 days. For experiments using *tub*-GAL80^ts^, flies were raised at 20°C and entrained under LD 12:12 cycles. The resulting pupae were placed in the eclosion monitors and the temperature raised to 28.5°C and kept at this temperature for the remainder of the experiment.

### Locomotor activity assays

Crosses (10 females + 5 males) were reared at 20°C under LD 12:12 light-dark cycle (lights-on at noon). Male progeny were collected under CO_2_ anesthesia on the day of eclosion, aged and entrained for 5–6 days, then placed in Trikinetics activity monitors. Their activity was then monitored under LD 12:12 conditions for 7–10 days followed by 7–10 days under DD conditions.

### Periodicity analyses

Periodicity of eclosion and locomotor activity records was analyzed using the Maximum Entropy Spectral Analysis (MESA) and Autocorrelation functions using the Matlab-based software from the “Fly Toolbox” software package [59]. The power of the eclosion and locomotor activity rhythms was determined using MESA analysis and the strength of rhythmicity was quantitated using the rhythmicity index (RI) derived from the Autocorrelation analysis, and categorized as rhythmic (RI≥0.3), weakly rhythmic (RI between 0.1–0.3) or arrhythmic (RI≤0.1 and obvious aperiodic records) [60].

### *In vivo* measurements of calcium and cAMP signals in the PG

#### Staging

Crosses were reared under 12h:12h LD conditions with lights-on at noon or midnight. For measurements made under DD conditions, cultures were wrapped in aluminum foil at lights-off and maintained covered until the desired time. Animals to be assayed were selected at the start of metamorphosis (white pre-pupal stage, WPP). Thus, developmental stage was kept constant; the only variable was time of day.

#### Measurements of calcium levels in the PG

Levels of calcium were estimated by measuring the fluorescence of the PG expressing the GCaMP3.2 sensor. For this, WPP stage animals were collected and the CNS (including the PG) dissected under ice-cold calcium-free fly saline (46 mM NaCl, 5 mM KCl, and 10 mM Tris pH 7.2). The CNS was mounted on slides coated with poly-lysine in an Attofluor chamber (A-7816, Invitrogen). PGs were imaged using an Olympus Spinning Disc microscope using an UMPlanFI 20X/0.50 water immersion objective and Cell^R (v. 2.6) program. Samples were visualized using the GFP filter (excitation 485nm and emission at 515nm) and imaged using a Hamamatsu camera (model ORCA IR2; 100 ms exposures).

#### Measurements of cAMP levels in the PG

cAMP levels in the PG were determined by measuring the FRET signal emitted by PGs expressing the *Epac1* sensor. The dissection protocol was the same as that used to measure calcium levels (above). Live FRET images were acquired using the same setup used for calcium, using a UMPlanFI 20X/0.50 water immersion objective, with exposure time of 100 ms. Images were analyzed using the Cell^R FRET v.2.6 program. Excitation of the CFP in *Epac1* sensor at a wavelength of 436nm led to YFP emission at 525nm but also CFP emission at 480nm [61]. Regions of interest (ROI) corresponding to individual PG cells were selected and background-subtracted CFP and YFP intensities were recorded. The ratio of YFP/CFP was determined after subtracting CFP spillover into the YFP channel using a recommended correction factor [62].

### Imaging of bioluminescence

Imaging was carried out as described in Selcho et al. (2017) [19]. Briefly, brains were dissected in ice-cold Schneider’s medium (Sigma-Aldrich) containing 1% antibiotic solution (10,000Uml^-1^ penicillin and 10 mg ml^-1^ streptomycin; Sigma-Aldrich), placed on poly-lysine coated FluoroDish plates (WPI, FL, USA). They were then covered with Schneider’s medium containing 1% antibiotic solution, supplemented with 10% fetal bovine serum and 10 mgml^-1^ insulin (Sigma-Aldrich), and containing 1mM luciferin (Potassium Salt, Gold Biotechnology Inc. MO, USA). Preparations were viewed using an LV200 microscope (Olympus, Japan) under 20X magnification and imaged for 72–96 h with an Evolve 512 camera (Photometrics, Tucson, AZ, USA) using 20min exposures and 200 X gain. Records were detrended using FIJI [63].

### Statistical analyses

Statistical analyses were carried out using Prism 6.0 (Graphpad Software Inc, CA). *t*-tests and one-way ANOVA, followed by Tukey’s *post hoc* multiple comparison analyses were used for normally distributed data; Wilcoxon rank sum tests were used for non-normally distributed data.

## Supporting information

S1 Fig Flies bearing single copies of UAS-RNAi insertions for genes in calcium homeostasis pathway express normal circadian rhythmicity of eclosion. (A) Average free-running period (h) values (± SEM) of flies heterozygous for UAS-RNAi transgenes for *cacophony*, *Ca-alpha1D*, *IP3* receptor (*IP3R*), *SERCA*, *CaMKII*, *CASK*, and for controls. Same letters indicate that there are no statistically significant differences between groups (one-way ANOVA, Tukey’s *post hoc* multiple comparison analyses); numbers in parenthesis indicate number of records averaged. (B) Average rhythmicity index (RI) values (± SEM) for results shown in A and for controls. Same letters indicate that there are no statistically significant differences between groups (one-way ANOVA, Tukey’s *post hoc* multiple comparison analyses); numbers in parenthesis indicate number of separate experiments.
(TIF)

S2 Fig Expression of GCaMP sensor does not affect free-running rhythmicity of eclosion and locomotor activity when expressed in the PG. (A) Representative records of adult emergence (left) and MESA analyses (right) of a population of animals expressing the GCaMP sensor in the PG (a), and in flies bearing only the *phm*-gal4 driver (b) or the UAS-GCaMP sensor (c). (B) Free-running periodicity values for genotypes shown in (A); each circle indicates results from separate experiments; average is indicated by horizontal line; same letters indicate no statistically different groups (one-way ANOVA, Tukey’s *post hoc* multiple comparison analyses). (C) Average rhythmicity index (RI) values (± SEM) for genotypes shown in (A); numbers in parenthesis indicate number of separate experiments; same letters indicate no statistically different groups (one-way ANOVA, Tukey’s *post hoc* multiple comparison analyses). (D) Representative records of adult locomotor activity in DD (left) and Autocorrelation analyses (right) of normal (*per*^*+*^) fly expressing GCaMP sensor in the PG (a) and of adult *per*^*01*^ fly expressing GCaMP sensor in the PG (b). (E) Free-running periodicity values for genotypes shown in (A); each circle indicates individual flies tested; average is indicated by horizontal line. (F) Corresponding average (± SEM) RI, ***p<0.0001 (two-tailed Student *t*-test with confidence interval of 95%). Numbers in parenthesis indicate total number of flies tested.
(TIF)

S3 Fig Flies bearing single copies of UAS-RNAi insertions for genes in cAMP pathway or of GAL4 drivers express normal circadian rhythmicity of eclosion. (A) Average free-running period (h) values (± SEM) of flies heterozygous for UAS-RNAi transgenes for *dunce*, *rutabaga*, *Epac*, *Rap1*, and *CREB*. Same letters indicate no statistically different groups (one-way ANOVA, Tukey’s *post hoc* multiple comparison analyses); numbers in parenthesis indicate number of records averaged. (B) Average rhythmicity index (RI) values (± SEM) for results shown in A. Same letters indicate no statistically different groups (one-way ANOVA, Tukey’s *post hoc* multiple comparison analyses); numbers in parenthesis indicate number of separate experiments.
(TIF)

S4 Fig Expression of *Epac1* sensor in the PG does not affect free-running rhythmicity of eclosion and locomotor activity when expressed in the PG. (A) Representative profiles and corresponding MESA analyses of timecourse of eclosion behavior of animals expressing *Epac*1 sensor in the PG (a); records of controls are shown below: *phm*-gal4 driver (b) and UAS-*Epac1* sensor alone (c). (B) Free-running periodicity values for genotypes shown in (A); each circle indicates results from separate experiments; average is indicated by horizontal line; same letters indicate no statistically different groups (one-way ANOVA, Tukey’s *post hoc* multiple comparison analyses). (C) Average rhythmicity index (RI) values (± SEM) for genotypes shown in (A); numbers in parenthesis indicate number of separate experiments; same letters indicate no statistically different groups (one-way ANOVA, Tukey’s *post hoc* multiple comparison analyses). (D) Representative records of adult locomotor activity in DD (left) and Autocorrelation analyses (right) of normal (*per*^*+*^) fly expressing *Epac1* sensor in the PG (a) and of *per*^*01*^ adult expressing *Epac1* sensor in the PG (b). (E) Free-running period values for results shown in (D); each circle indicates individual flies tested; average is indicated by horizontal line. (F) Average (± SEM) RI, ***p<0.0001 (two-tailed Student *t*-test with confidence interval of 95%). Numbers in parenthesis indicate total number of flies tested.
(TIF)
