# Supplementary figures and images for: Calcium and cAMP directly modulate the speed of the Drosophila circadian clock
Source: PLoS Genet. 2018 Jun 7;14(6):e1007433. doi: 10.1371/journal.pgen.1007433 (PMC6007936; doi:10.1371/journal.pgen.1007433)

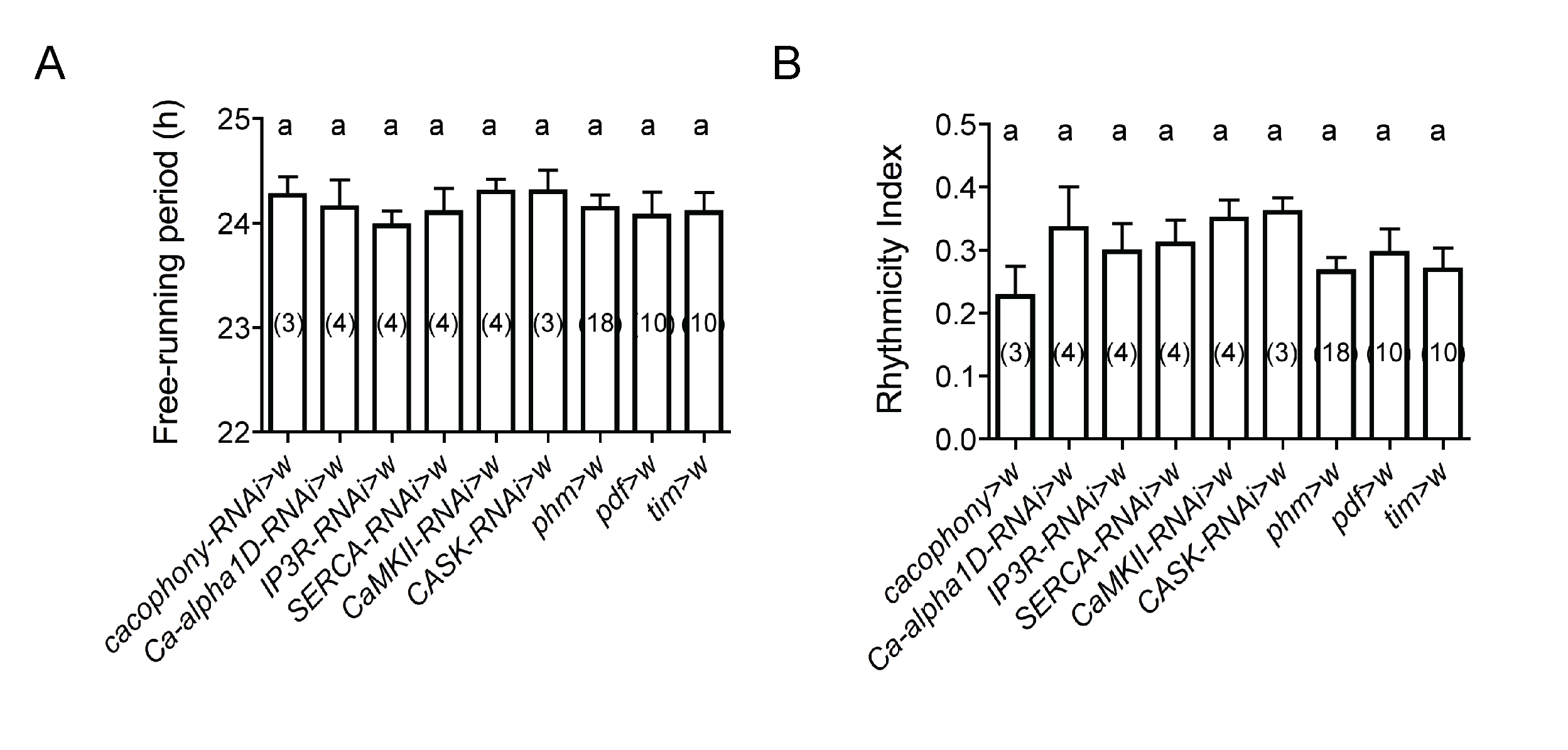

Supplement: S1 Fig — (A) Average free-running period (h) values (± SEM) of flies heterozygous for UAS-RNAi transgenes for cacophony, Ca-alpha1D, IP3 receptor (IP3R), SERCA, CaMKII, CASK, and for controls. Same letters indicate that there are no statistically significant differences between groups (one-way ANOVA, Tukey’s post hoc multiple comparison analyses); numbers in parenthesis indicate number of records averaged. (B) Average rhythmicity index (RI) values (± SEM) for results shown in A and for controls. Same letters indicate that there are no statistically significant differences between groups (one-way ANOVA, Tukey’s post hoc multiple comparison analyses); numbers in parenthesis indicate number of separate experiments. (TIF) [file pgen.1007433.s001.tif]

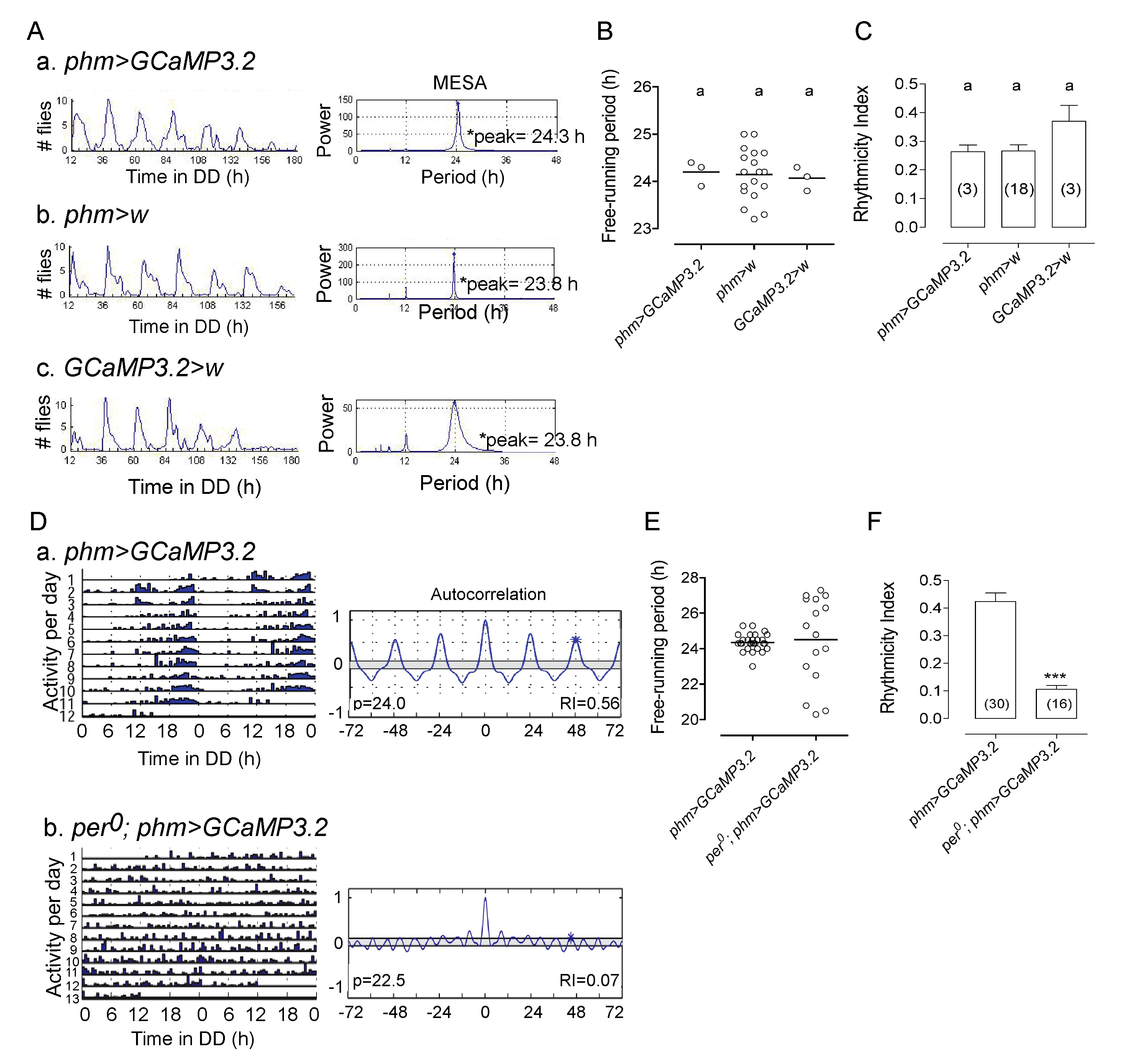

Supplement: S2 Fig — (A) Representative records of adult emergence (left) and MESA analyses (right) of a population of animals expressing the GCaMP sensor in the PG (a), and in flies bearing only the phm-gal4 driver (b) or the UAS-GCaMP sensor (c). (B) Free-running periodicity values for genotypes shown in (A); each circle indicates results from separate experiments; average is indicated by horizontal line; same letters indicate no statistically different groups (one-way ANOVA, Tukey’s post hoc multiple comparison analyses). (C) Average rhythmicity index (RI) values (± SEM) for genotypes shown in (A); numbers in parenthesis indicate number of separate experiments; same letters indicate no statistically different groups (one-way ANOVA, Tukey’s post hoc multiple comparison analyses). (D) Representative records of adult locomotor activity in DD (left) and Autocorrelation analyses (right) of normal (per+) fly expressing GCaMP sensor in the PG (a) and of adult per01 fly expressing GCaMP sensor in the PG (b). (E) Free-running periodicity values for genotypes shown in (A); each circle indicates individual flies tested; average is indicated by horizontal line. (F) Corresponding average (± SEM) RI, ***p<0.0001 (two-tailed Student t-test with confidence interval of 95%). Numbers in parenthesis indicate total number of flies tested. (TIF) [file pgen.1007433.s002.tif]

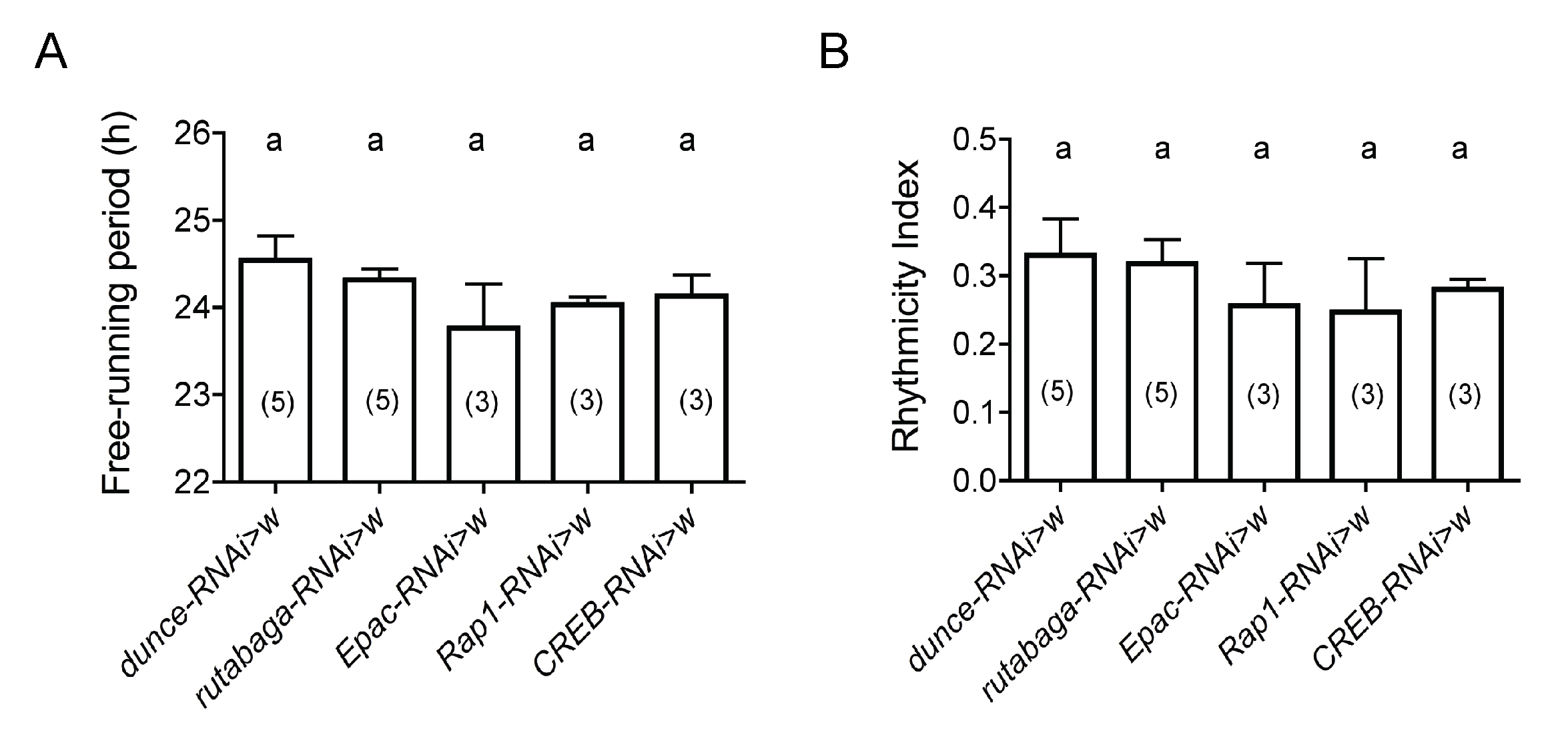

Supplement: S3 Fig — (A) Average free-running period (h) values (± SEM) of flies heterozygous for UAS-RNAi transgenes for dunce, rutabaga, Epac, Rap1, and CREB. Same letters indicate no statistically different groups (one-way ANOVA, Tukey’s post hoc multiple comparison analyses); numbers in parenthesis indicate number of records averaged. (B) Average rhythmicity index (RI) values (± SEM) for results shown in A. Same letters indicate no statistically different groups (one-way ANOVA, Tukey’s post hoc multiple comparison analyses); numbers in parenthesis indicate number of separate experiments. (TIF) [file pgen.1007433.s003.tif]

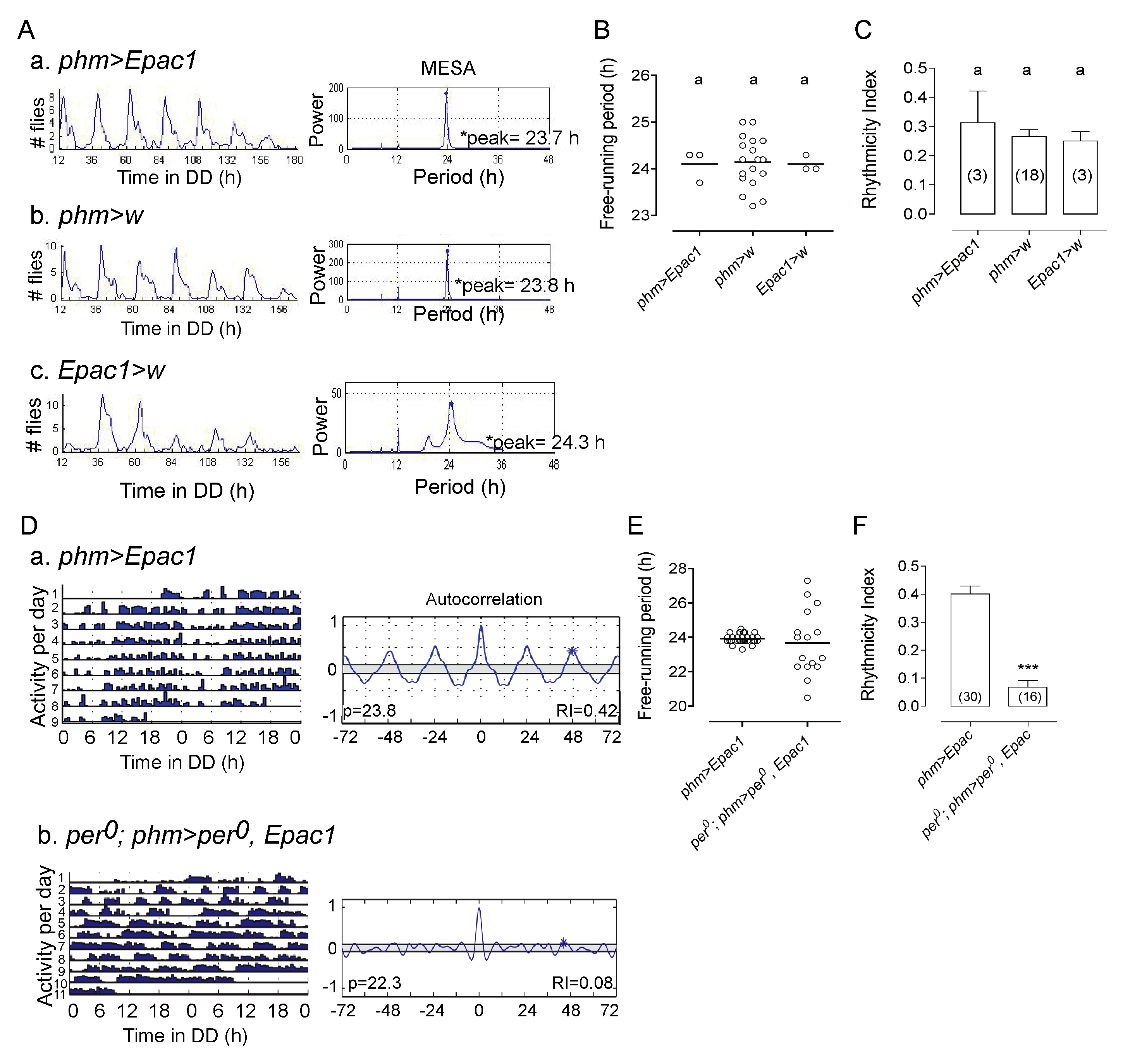

Supplement: S4 Fig — (A) Representative profiles and corresponding MESA analyses of timecourse of eclosion behavior of animals expressing Epac1 sensor in the PG (a); records of controls are shown below: phm-gal4 driver (b) and UAS-Epac1 sensor alone (c). (B) Free-running periodicity values for genotypes shown in (A); each circle indicates results from separate experiments; average is indicated by horizontal line; same letters indicate no statistically different groups (one-way ANOVA, Tukey’s post hoc multiple comparison analyses). (C) Average rhythmicity index (RI) values (± SEM) for genotypes shown in (A); numbers in parenthesis indicate number of separate experiments; same letters indicate no statistically different groups (one-way ANOVA, Tukey’s post hoc multiple comparison analyses). (D) Representative records of adult locomotor activity in DD (left) and Autocorrelation analyses (right) of normal (per+) fly expressing Epac1 sensor in the PG (a) and of per01 adult expressing Epac1 sensor in the PG (b). (E) Free-running period values for results shown in (D); each circle indicates individual flies tested; average is indicated by horizontal line. (F) Average (± SEM) RI, ***p<0.0001 (two-tailed Student t-test with confidence interval of 95%). Numbers in parenthesis indicate total number of flies tested. (TIF) [file pgen.1007433.s004.tif]
